# Lipoprotein(a) and High Sensitivity C-Reactive Protein among Patients with HIV in Ghana: The Study on Cardiovascular Risk Profile of HIV-Infected Patients on HAART (SCRIPT)

**DOI:** 10.5334/gh.850

**Published:** 2020-11-03

**Authors:** Lambert Tetteh Appiah, Fred Stephen Sarfo, Samuel Blay Nguah, Mark D. Huffman, Jonathan K. Stiles, Matthew J. Feinstein

**Affiliations:** 1 Komfo Anokye Teaching Hospital, Kumasi, GH; 2 Kwame Nkrumah University of Science andTechnology, School of Medicine and Dentistry, Kumasi, GH; 3 Northwestern University Feinberg School of Medicine, Chicago, US; 4 The George Institute for Global Health, Sydney, AU; 5 Morehouse School of Medicine, Atlanta, US

**Keywords:** lipoprotein(a), high sensitivity C-reactive protein, cardiovascular disease (CVD), CVD risk, antiretroviral therapy, Ghana

## Abstract

**Background:**

Lipoprotein(a) [Lp(a)] and high-sensitivity C-reactive protein (hs-CRP) levels are associated with cardiovascular disease (CVD) in the general population, even after adjusting for conventional CVD risk factors. However, data are limited regarding the distribution of Lp(a) and hs-CRP among patients with HIV in Ghana. We explored levels of Lp(a), hs-CRP and other cardiovascular risk factors among people who were HIV positive (HIV+) on ART (HIV+ART+), HIV+ART–, and HIV–ART– in a Ghanaian population.

**Methods:**

We conducted a cross sectional study, recruited individuals who were HIV+ART+ and HIV+ART– from the largest HIV clinic in central Ghana between August 2018 and December 2019. HIV negative controls were recruited from communities and adjoining suburbs of Kumasi. Lipoprotein(a) was measured using Immunoturbidimetric assay and high sensitive-CRP concentrations were determined using particle-enhanced turbidimetric assay. We compared levels of Lp(a), hs-CRP, and conventional CVD risk factors among these groups and used multivariable stepwise logistic regression models to explore associations between them.

**Results:**

Among HIV+ART+ (n = 156), HIV+ART– (n = 131), and HIV–ART– (n = 147), mean(SD) ages were 48 (9.1) years, 41 (11.1) years and 45 (11.9) years, p = <0.001, proportion of females were 71.2%, 67.9% and 73.5% respectively. Median(IQR) concentrations of hs-CRP in mg/L were 1.7 (0.8,4.5), 2.03 (0.5,8.58) and 1.0 (0.45,2.74) across respective groups and the proportion of elevated Lp(a) concentrations (Lp[a] > 30mg/dL) were 70%, 48% and 62% among HIV+ART+,HIV+ART– and HIV–ART– participants respectively. Diabetes mellitus, dyslipidemia, waist-to-hip ratio and metabolic syndrome were associated with higher hs-CRP levels. Compared to HIV–ART–, HIV+ patients had higher odds of having hs-CRP > 3mg/L while HIV+ART+ patients had higher odds of elevated Lp(a) than HIV+ART– after multivariable adjustment.

**Conclusion:**

PLWHA in Ghana are associated with higher odds of elevated hs-CRP, regardless of ART use. HIV+ART+ is significantly associated with higher odds of elevated Lp(a) levels compared to HIV+ART–; even after multivariable adjustments. Reasons for this and potential clinical implications merit further study.

## Introduction

Sub-Saharan Africa (SSA) has seen a dramatic improvement in the life expectancy and quality of life of people living with HIV/AIDS (PLWHA) with the advent, scale-up, and sustained use of effective antiretroviral therapy (ART) since 2004 [1]. HIV infection has thus evolved to become a manageable chronic illness. With the aging of PLWHA and prolonged use of ART, PLWHA have become more susceptible to non-AIDS co-morbidities, such as cardiovascular diseases (CVDs), even as AIDS-related mortality steadily declines [2,3]. Non-AIDS-related complications occur approximately 10 years earlier among PLWHA compared to the general population, suggesting an acceleration of the ageing process in the course of the infection [4]. This may result from the complex interplay between HIV-related factors, including immune dysregulation and inflammation, and conventional and emerging risk factors for common diseases, including cardiovascular disease (CVD).

Conventional CVD risk factors have been studied and found to be prevalent among both HIV infected patients and the general population in Ghana [5,6,7]. In this context, lipoprotein(a) [Lp(a)] and high sensitivity C-reactive protein (hs-CRP) are of interest given their associations with CVD risk and their roles in pro-inflammatory atherogenic dyslipidemia and general inflammation, respectively. Lp(a) particles are a sub-class of low density lipoproteins(LDL) primarily distinguished by their proatherogenic and prothrombotic apolipoprotein(a)[apo(a)] component. Elevated lipoprotein(a),though predominantly determined by genetic factors [8], is an established independent atherosclerotic CVD risk factor described in the general population akin to conventional LDL cholesterol(LDL-C). Race or ethnicity is an important variable influencing Lp(a) levels [9] and hence cutoffs for CVD risk assessment. Several studies including the Justification for the Use of Statins in primary prevention: An intervention Trial Evaluating Rosuvastatin (JUPITER) trial have shown that blacks have a 2–3 fold higher median Lp(a) levels relative to Caucasians [10]. Despite these racial discrepancies, the Multi-ethnic Study of Atherosclerosis (MESA) has demonstrated that the 30mg/dL Lp(a) cut off remains suitable for blacks unlike whites in predicting coronary heart disease risk [11]. Similarly, hs-CRP, a biomarker of generalized inflammation, has been established as an independent predictor of CVDs even after adjusting for other established CVD risk factors [12]. Whether this association exists of CRP as a marker of cardiovascular risk among HIV-infected patients remains unclear given the conflicting reports from the limited available data in this setting [13,14].

We have previously described the burden of CVD risk factors among PLWHA in Ghana [5]; however, there are limited data in the West African sub-region on these emerging CVD risk factors with little data on lipoprotein(a) and how it relates to other traditional CVD risk factors. This **S**tudy on **C**ardiovascular **Ri**sk **P**rofile of HIV-infected patients on HAAR**T** (SCRIPT) sought to describe the distributions of lipoprotein(a) and hs-CRP in the context of traditional CVD risk factors at the second largest teaching hospital in Ghana.

## Methods

### Study Setting and Recruitment

The study recruited PLWHA who were 18 years and older and who received care at the Komfo Anokye Teaching Hospital (KATH) between August 2018 and December 2019. KATH is a 1,200-bed facility in Kumasi and the second-largest teaching hospital in Ghana, serving approximately 4 million people in the Ashanti Region. KATH is affiliated with The School of Medicine and Dentistry of the Kwame Nkrumah University of Science and Technology (KNUST). KATH’s HIV clinic cares for approximately 6,000 patients annually and is the largest HIV clinic within central and northern Ghana. The hospital offers general and specialized medical care and serves as a referral hospital for all regional and district hospitals in the Ashanti region and to 10 out of 16 other regions across country. This study was approved by the Committee on Human Research, Publication and Ethics (CHRPE) of the Kwame Nkrumah University of Science and Technology, School of Medicine and Dentistry (KNUST-SMD) with reference number CHRPE/AP/509/18.

We enrolled consecutive adult HIV patients 18 years or older presenting to the HIV clinic for routinely scheduled visits and HIV-negative adults from communities and adjoining suburbs of the metropolitan city of Kumasi to serve as controls. A community leader was contacted at each community, informed about the project to gain Community entrance for this study. A designated community center was selected, an announcement was made on an agreed date(s) and eligible participants were consecutively screened and those who meet entry criteria were recruited by trained research personnel. All participants provided written informed consent.

### Study Procedures

All participants completed a standardized questionnaire to capture data on demographics, socioeconomic status, medical history, and medication use by trained study personnel. At the time of interview, anthropometry, blood pressure, and ankle brachial pressure index (ABI) were obtained. Weight and height were measured without shoes while wearing light clothes, and body mass index was calculated as the weight in kilograms divided by the square of the height in meters. The abdominal circumference was measured as the narrowest circumference between the lower rib margin and anterior superior iliac crest above the umbilicus at exhalation. Participants had their systolic blood pressure (SBP) and diastolic blood pressure (DBP) measured after five minutes of rest in a seated position with their arms, back, and feet supported. The first and fifth Korotkoff sounds were registered to indicate SBP and DBP, respectively. Two blood pressure measures were obtained, and the mean was calculated. The Ankle-Brachial-Index(ABI) was measured for each participant following a five-minute rest in the supine position using the Boso ABI device. A pressure cuff was placed on either arm and then above the ankles. The ABI value on either the left or right side of the body was determined as the ratio of the ankle systolic blood pressure to the brachial systolic blood pressure on that side.

Blood samples were obtained from all participants through venipuncture. Serum concentrations of HbA1c, total cholesterol, HDL cholesterol and triglycerides were determined using the enzymatic method in the Cobas Integra 400 (Roche). Lipoprotein(a) and hs-CRP concentrations were determined using the Immunoturbidimetric assay and the particle-enhanced turbidimetric assays respectively and standardized against their respective WHO references. CD4 lymphocyte count was performed using Becton Dickinson FACSCalibur flow cytometer 4 color basic and viral load testing was carried out using the Cobas AmpliPrep, Cobas Taqman 94 polymerase reaction test machine.

### Study Definitions

Body mass index (BMI) of less than 18.5 kg/m^2^ defined underweight, 18.5–24.9 kg/m^2^ defined normal body weight, 25–29.9 kg/m^2^ defined overweight, and equal to or greater than 30 kg/m^2^ defined obesity. Truncal obesity was defined as waist-to-hip ratio of ≥0.9 in men and ≥0.85 in women [15]. Current or past smoking history was ascertained from participants. Hypertension was defined as SBP ≥ 140 mm Hg, DBP ≥ 90 mm Hg, or self-reported use of blood pressure lowering medication. Stages 1 and 2 hypertension groups were classified according to JNC 7 guidelines [16]. Hypercholesterolemia was defined as total cholesterol ≥200 mg/dL (≥5.18 mmol/L) or self-reported use of lipid lowering therapy. Hypertriglyceridemia was defined as triglycerides ≥150 mg/dL (≥1.7 mmol/L). Low HDL cholesterol was defined as HDL-C ≤50 mg/dL (≤1.30 mmol/L) for women or ≤40 mg/dL (≤1.04 mmol/L) for men. High LDL cholesterol was defined as LDL-C ≥150 mg/dL (≥3.8 mmol/L). Diabetes mellitus was defined as a previous diagnosis of type 1 or 2 diabetes mellitus, at least two random blood glucose readings of ≥11.1 mmol/L, fasting plasma glucose reading of ≥7 mmol/L, or self-reported use of a glucose-lowering agent. Peripheral artery disease (PAD) was defined as an ABI of ≤0.9 in either limb [17]. Elevated Lp(a) and elevated hs-CRP levels were defined as Lp(a) ≥ 30mg/dl and hs-CRP > 3mg/L respectively [18,19].

### Statistical Analysis

We first compared the demographics and clinical covariates of individuals with HIV infection on ART (HIV+ART+), HIV infection not on ART (HIV+ART–) and healthy adults without HIV (HIV–ART–) using either ANOVA or Kruskal Wallis test for continuous covariates. Subsequently, we compared the levels of Lp(a), hs-CRP and the traditional CVD risk factors. For categorical risk factors, the test for association was done using a chi-square test, if none of the counts was less than five, and Fisher’s exact test otherwise. Continuous clinical covariates with approximately normal distribution were analyzed for difference in means using the two-tailed t-test. For skewed data, the Mann-Whitney U test was used. To determine the association between Lp(a) and hs-CRP and HIV and ART status, multivariable logistic regression models were used. Model 1 tested the association between hs-CRP or LP(a) as dependent variables and HIV positive only or HAART only as predictors. Model 2 tested the association between hs-CRP or LP(a) as dependent variables and HIV positive and HAART as predictors. Model 3 tested the association between hs-CRP or LP(a) as dependent variables and HIV positive, HAART, age and sex as predictors. Model 4 tested the association hs-CRP or LP(a) as dependent variables and HIV positive, HAART, age, sex, blood pressure, dyslipidemia, diabetes mellitus, waist-hip ratio, tobacco use, metabolic syndrome and body mass index as predictors. Finally Model 5 tested hs-CRP or LP(a) as dependent variables and HIV positive, HAART, age, sex, blood pressure, dyslipidemia, diabetes mellitus, waist-hip ratio, tobacco use, metabolic syndrome, body mass index and either hs-CRP or Lp(a) as predictors. In all analyses, two-sided p-values of <0.05 were considered statistically significant. We used Stata SE (version 15, College Station, TX) for statistical analyses.

## Results

### Characteristics of study population

**Figure 1:**
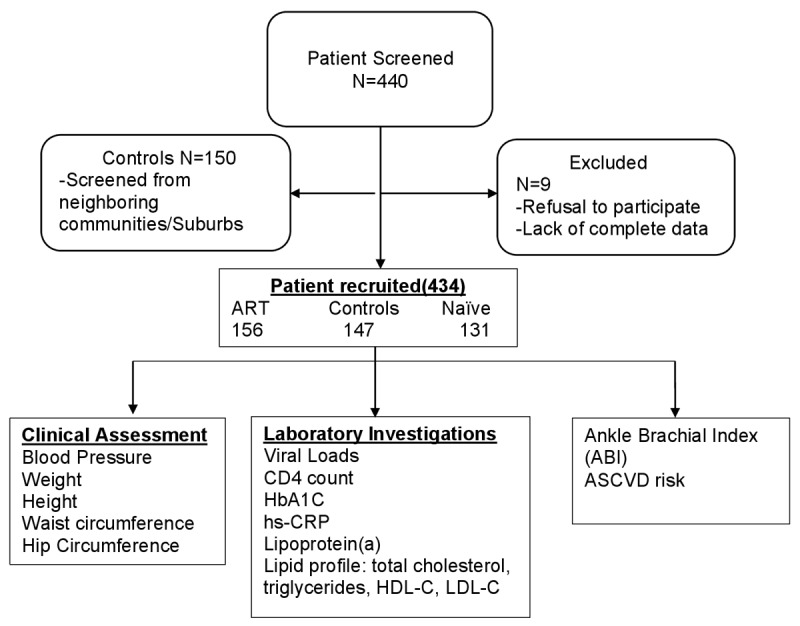
Flow Chart of SCRIPT stydy participants. ASCVD; atherosclerotic cardiovascular disease

Figure 1 shows the flow chart for study participants and baseline study characteristics in Table 1. The analysis included 156 HIV patients receiving ART (HIV+ART+), 131 HIV patients not receiving ART (HIV+ART–), and 147 participants without HIV (HIV–ART–). There were 111 (71.2%), 89 (68%), and 108 (73.5%) females among HIV+ART+, HIV+ART–, and HIV–ART– groups, respectively. The mean (±SD) age of HIV+ART+ was 48 (±9.1) years, HIV+ART– was 41 (±11.1) years, and HIV–ART– was 45 (±11.9) years. Most participants were married (57.4%), non-tobacco users (92.2%), never used alcohol (61.1%), and 49.0% had a monthly income of less than 500 Ghana cedis ($100). Seventy percent of HIV+ART+ had undetectable viral loads with a median (IQR) CD4 count of 623 (379,867) compared to only 6.5% viral load suppression with a median (IQR) CD4 count of 253 (112,415) among HIV+ART– participants.

**Table 1:** Baseline characteristics of study population.

		HIV–ART– (n = 147)	HIV+ART– (n = 131)	HIV+ART+ (n = 156)	All (n = 434)	P-value

Age, years, mean (SD)		45 (11.9)	41 (11.1)	48 (9.1)	45 (11.0)	<0.001
Female, n (%)		108 (73.5)	89 (67.9)	111 (71.2)	308 (71.0)	0.597
Marital status, n (%)	Married	108 (73.5)	68 (51.9)	73 (46.8)	249 (57.4)	<0.001
	Single	38 (25.9)	53 (40.5)	59 (37.8)	150 (34.6)	
	Unknown	1 (0.7)	10 (7.6)	24 (15.4)	35 (8.1)	
Education	None	10 (6.8)	17 (13.0)	25 (16.0)	52 (12.0)	0.001
	Primary	85 (57.8)	84 (64.1)	106 (67.9)	275 (63.4)	
	Secondary	31 (21.1)	16 (12.2)	21 (13.5)	68 (15.7)	
	Tertiary	21 (14.3)	14 (10.7)	4 (2.6)	39 (9.0)	
Individual monthly income (GHC)	<500	78 (53.1)	61 (46.6)	72 (46.2)	211 (48.6)	0.179
	500–1000	37 (25.2)	41 (31.3)	38 (24.4)	116 (26.7)	
	>1000	13 (8.8)	7 (5.3)	23 (14.7)	43 (9.9)	
WHO stage, n (%)	Stage I	–	61 (46.6)	58 (37.2)	119 (41.5)	0.010
	Stage II	–	10 (7.6)	24 (15.4)	34 (11.8)	
	Stage III	–	58 (44.3)	62 (39.7)	120 (41.8)	
	Stage IV	–	2 (1.5)	12 (7.7)	14 (4.9)	
Tobacco Use, n (%)	Current User	0 (0)	8 (6.1)	4 (2.6)	12 (2.8)	0.007
	Previous user	4 (2.7)	7 (5.3)	11 (7.1)	22 (5.1)	
	Never Used	143 (97.3)	116 (88.5)	141 (90.4)	400 (92.2)	
Alcohol use, n (%)	Current User	19 (12.9)	27 (20.6)	15 (9.6)	61 (14.1)	<0.001
	Previous user	16 (10.9)	39 (29.8)	53 (34)	108 (24.9)	
	Never Used	112 (76.2)	65 (49.6)	88 (56.4)	265 (61.1)	
Medications, n (%)	Antihypertensives	19 (12.9)	14 (10.7)	28 (17.9)	61 (14.1)	0.188
	Anti-diabetics	4 (2.7)	6 (4.6)	2 (1.3)	12 (2.8)	0.237
	Statin	2 (1.4)	0 (0)	0 (0)	2 (0.5)	0.205
	Aspirin	1 (0.7)	3 (2.3)	4 (2.6)	8 (1.8)	0.446
Current CD4 counts, unit, median, IQR			253 (111–414)	623 (378–867)	401 (206–667)	<0.001
Months since HIV diagnosis, median, IQR			1 (0–2)	131 (83–158)	58 (0.8–136)	<0.001
Suppressed viremia, n (%)			7 (6.5)	104 (70.3)	111 (43.5)	<0.001
Log Viral load in unsuppressed, units, mean (SD)			11.5 (2.1)	8.4 (3.1)	10.5 (2.8)	<0.001
Current ART regimen, n (%)	TDF + 3TC + EFZ			53 (34.0)	53 (34.0)	NA
	AZT + 3TC + NVP			39 (25.0)	39 (25.0)	
	AZT + 3TC + EFZ			34 (21.8)	34 (21.8)	
	TDF + 3TC + LPV/r			11 (7.1)	11 (7.1)	
	TDF + 3TC + NVP			7 (4.5)	7 (4.5)	
	AZT+ 3TC			2 (1.3)	2 (1.3)	
	AZT + NVP			2 (1.3)	2 (1.3)	
	Other Regimen			8 (5.1)	8 (5.1)	

HIV+ART+; HIV patient on anti-retroviral therapy(ART), HIV+ART–; HIV patient not on treatment, HIV–ART–; HIV uninfected individual, WHO stage; World health Organization clinical staging of HIV infection, CD4; cluster of differentiation four T-lymphocytes, TDF, tenofovir; 3TC, Lamivudine; NVP, Nevirapine; AZT, Zidovudine; EFZ, Efavirenz, LPV/r, Lopinavir with low dose ritonavir.

### Cardiovascular Risk Factors

Table 2 presents the baseline cardiovascular risk factors. The mean (±SD) BMI of HIV+ART+ was 25.2 (±5.6) kg/m^2^, HIV+ART– was 23.1 (±4.2) kg/m^2^ and 27.4 (±5.1) kg/m^2^ among HIV–ART– participants, p =< 0.001. The mean SBP (±SD) was 130.6 (±23.6) mmHg, 120.9 (±22.6) mmHg and134.5 (±22.8) mmHg among HIV+ART+, HIV+ART– and HIV–ART– participants respectively, p = <0.001. Diabetes prevalence was 4.5%, 6.1% and 7.5% among HIV+ART+, HIV+ART– and HIV–ART– respectively. The median(IQR) hs-CRP concentration among HIV+ART+ was 1.7 (0.80,4.50) mg/L, HIV+ART– was 2.03 (0.50,0.58) mg/L and among HIV–ART– was 1.0 (0.45,2.74) mg/L, p = 0.003. Elevated Lp(a) concentrations were seen in 70% of HIV+ART+, 48.3% of HIV+ART– and 62.4% among HIV–ART– study participants. Similarly, across the respective groups, dyslipidemia was present in 76%, 84% and 75% respectively. The median(IQR) ASCVD risk score was 2.7 (0.9,6.6)% in HIV+ART+, 2.1 (0.5,4.3)% in HIV+ART– and 2.8 (0.6,5.2) among HIV–ART– participants, p = 0.115.

**Table 2:** Baseline cardiovascular risk factors.

Risk Factor		HIV–ART– (n = 147)	HIV+ART– (n = 131)	HIV+ART+ (n = 156)	All (n = 434)	P-value

BMI kg/m^2^ mean (SD)		27.4 (5.1)	23.1 (4.2)	25.2 (5.6)	25.3 (5.3)	<0.001
Waist circumference (cm) mean (SD)		90 (16.6)	82 (8.7)	87 (11.7)	87 (13.3)	<0.001
Waist-Hip-Ratio mean (SD)		0.888 (0.071)	0.893 (0.067)	0.904 (0.071)	0.895 (0.070)	0.151
SBP (mmHg) mean (SD)		134.5 (22.8)	120.9 (22.6)	130.6 (23.6)	129.0 (23.0)	<0.001
DBP (mmHg) mean (SD)		82.6 (15.5)	80.2 (14.1)	84.4 (14.5)	82.5 (14.8)	0.051
Hypertension pharmacotherapy with control, n (%)		12 (17.6)	12 (27.9)	16 (21.6)	40 (21.6)	0.442
Self-reported comorbidities (n, %)	Hyperlipidemia	9 (6.1)	2 (1.5)	4 (2.6)	15 (3.5)	0.104
	Hypertension	25 (17)	17 (13)	41 (26.3)	83 (19.1)	0.012
	Diabetes	6 (4.1)	6 (4.6)	2 (1.3)	14 (3.2)	0.205
	Stroke	0 (0)	3 (2.3)	7 (4.5)	10 (2.3)	0.018
	Myocardial infarction	0 (0)	1 (0.8)	4 (2.6)	5 (1.2)	0.108
	Heart Failure	0 (0)	0 (0)	1 (0.6)	1 (0.2)	1.000
	PAD	2 (1.4)	53 (40.5)	57 (36.5)	112 (25.8)	<0.001
Self-reported family history (n,%)	Hyperlipidemia	2 (1.4)	1 (0.8)	3 (1.9)	6 (1.4)	0.877
	Hypertension	52 (35.4)	36 (27.5)	54 (34.6)	142 (32.7)	0.308
	Diabetes	23 (15.6)	20 (15.3)	25 (16.0)	68 (15.7)	0.985
	Stroke	8 (5.4)	10 (7.6)	16 (10.3)	34 (7.8)	0.295
Hemoglobin A1c percentage mean (SD)		5.6 (0.9)	5.4 (0.6)	5.3 (0.6)	5.4 (0.7)	<0.001
Diabetes mellitus, % (n, %)		11 (7.5)	8 (6.1)	7 (4.5)	26 (6)	0.546
Hs-CRP (mg/L) median (IQR)		1.00 (0.45–2.74)	2.03 (0.50–8.58)	1.70 (0.80–4.50)	1.60 (0.50–4.10)	0.003
Lipoprotein(a) (n, %)	≥30mg/dL	83 (62.4)	58 (48.3)	108 (69.7)	249 (61.0)	0.001
	<30mg/dL	50 (37.6)	62 (51.7)	47 (30.3)	159 (39.0)	
LDL-Cholesterol, mmol/L, mean (SD)		3.35 (1.07)	2.75 (0.9)	3.22 (0.99)	3.12 (1.02)	<0.001
LDL-Cholesterol ≥ 3.4 mmol/l (n, %)		64 (47.8)	34 (27.4)	64 (41.8)	162 (39.4)	0.003
HDL-Cholesterol, mmol/L, mean (SD)		1.36 (0.29)	1.02 (0.36)	1.41 (0.33)	1.28 (0.37)	<0.001
HDL-Cholesterol ≤ 1.03 mmol/l		42 (31.3)	85 (68.5)	43 (27.7)	170 (41.2)	<0.001
Triglyceride, mmol/L median (IQR)		1.05 (0.7–1.6)	1.17 (0.9–1.66)	1.29 (0.92–1.78)	1.17 (0.84–1.72)	0.031
Triglyceride ≥ 1.7 mmol/l		31 (23.1)	29 (23.4)	47 (30.3)	107 (25.9)	0.283
Dyslipidemia, n (%)		100 (74.6)	104 (83.9)	118 (76.1)	322 (78.0)	0.158
10-year predicted atherosclerotic cardiovascular disease risk score, median (IQR)		2.8 (0.6–5.2)	2.1 (0.5–4.3)	2.7 (0.9–6.6)	2.6 (0.6–5.5)	0.115

HIV+ART+; HIV patient on anti-retroviral therapy(ART), HIV+ART–; HIV patient not on treatment, HIV–ART–; HIV uninfected individual. Diabetes mellitus defined as a previous diagnosis of type 1 or 2 diabetes mellitus, at least 2 random blood glucose readings of ≥11.1 mmol/L, fasting plasma glucose reading of ≥7 mmol/L, or self-reported use of a glucose-lowering agent.

### Predicted Cardiovascular Risk by Lp(a) and hs-CRP

As seen in Table 3, there was no difference in the means of the systolic blood pressures across the various concentrations of both Lp(a) and hs-CRP. Elevated Lp(a) however, appears to be associated with a higher mean diastolic blood pressure, a higher mean total cholesterol and a higher mean LDL-C levels. High hs-CRP levels are associated with a higher median triglyceride levels and a higher median waist-to-hip ratio. Table 3 again shows that diabetes mellitus, dyslipidemia and metabolic syndrome were associated with higher hs-CRP levels (>3mg/L).

**Table 3:** Distribution of cardiovascular risk per levels of Lp(a) and hs-CRP.

Risk factor	Lipoprotein(a)			hs-CRP		

	<30mg/dl	≥30mg/dl	P-value	<3mg/L	≥3mg/L	P-value

Sample size	n = 159	n = 249		n = 282	n = 128	

Systolic BP, mean (SD)	126.9 (23.1)	130.1 (23.6)	0.176	130.1 (23.6)	126.8 (23.9)	0.200
Diastolic BP, mean (SD)	80.4 (13.8)	83.5 (15.4)	0.041	82.4 (15.3)	82.6 (14.3)	0.902
Proportion with HPT, n (%)	61 (38.4)	113 (45.4)	0.162	116 (41.1)	61 (47.7)	0.217
Proportion of HPT on Rx, n (%)	20 (32.8)	37 (32.7)	0.995	34 (29.3)	23 (37.7)	0.256
Controlled on HPT Rx, n (%)	11 (18.0)	26 (23.0)	0.444	19 (16.4)	19 (31.2)	0.023
Hemoglobin A1c, mean (SD)	5.37 (0.56)	5.50 (0.80)	0.088	5.42 (0.70)	5.52 (0.74)	0.162
Proportion with DM	7 (4.4)	17 (6.8)	0.310	10 (3.6)	14 (11.0)	0.003
Total cholesterol, mean (SD)	4.7 (1.2)	5.3 (1.3)	<0.001	5.3 (1.2)	5.4 (1.5)	0.956
LDL-C, mean (SD)	2.8 (0.9)	3.3 (1.0)	<0.001	3.1 (1.0)	3.2 (1.1)	0.304
HDL-C, mean (SD)	1.21 (0.38)	1.32 (0.35)	0.003	1.34 (0.33)	1.13 (0.42)	<0.001
Triglyceride, median (IQR)	1.2 (0.8,1.8)	1.2 (0.9,1.7)	0.874	1.1 (0.8,1.6)	1.5 (1.0,1.9)	<0.001
Dyslipidemia, n (%)	119 (74.8)	199 (80.0)	0.228	203 (72.0)	117 (91.4)	<0.001
Current Cigarette use	4 (2.5)	8 (3.2)	0.863	7 (2.5)	4 (3.1)	0.456
Previous Cigarette Use	9 (5.7)	12 (4.8)		12 (4.3)	9 (7.0)	
Body Mass Index, mean (SD)	25.2 (5.6)	25.5 (5.3)	0.610	25.1 (4.7)	26.0 (6.7)	0.105
Waist-to-Hip ratio, median (SD)	0.90 (0.06)	0.89 (0.07)	0.479	0.88 (0.07)	0.91 (0.06)	0.002
Peripheral Artery Disease						
Normal	63 (37.1)	107 (62.9)	0.287	126 (74.1)	44 (25.9)	0.387
0.81–0.9 (Mild)	27 (40.3)	40 (59.7)		42 (63.6)	24 (36.4)	
0.5–0.8 (moderate)	15 (25)	45 (75)		40 (66.7)	20 (33.3)	
≤0.49 (severe)	4 (33.3)	8 (66.7)		8 (66.7)	4 (33.3)	
Metabolic syndrome, n (%)	63 (39.6)	92 (37.0)	0.587	92 (32.6)	65 (50.8)	<0.001
Study group						
Control	50 (37.6)	83 (62.4)	0.001	106 (79.7)	27 (20.3)	0.001
Naïve	62 (51.7)	58 (48.3)		72 (58.5)	51 (41.5)	
ART	47 (30.3)	108 (69.7)		104 (67.5)	50 (32.5)	

Compared to HIV–ART–, HIV+ patients overall had higher odds of having hs-CRP > 3mg/L after adjusting for age, sex, and other traditional CVD risk factors (Table 4). Compared to HIV–, HIV+ participants were associated with higher odds of an elevated hs-CRP concentration even after adjustment for age, sex, cardiovascular risk factors. Across models, individuals who were HIV+ART+ similarly were associated with higher odds of elevated Lp(a) than HIV+ART– after multivariable adjustment.

**Table 4:** Factors associated with high concentrations of Lp(a) and hs-CRP.

HIV Positive	Lipoprotein(a) ≥ 30mg/dl		Hs-CRP (>3mg/L)	

	OR (95%CI)	p-Value	OR (95%CI)	p-Value

Model 1	0.92 (0.60–1.40)	0.025	2.25 (1.38–3.67)	<0.001
Model 2	0.56 (0.34–0.93)	0.024	2.78 (1.60–4.84)	<0.001
Model 3	0.58 (0.35–0.97)	0.039	3.19 (1.80–5.65)	<0.001
Model 4	0.44 (0.24–0.80)	0.006	3.31 (1.68–6.52)	<0.001
Model 5	0.46 (0.25–0.84)	0.012	3.34 (1.67–6.67)	<0.001
**On ART**	**OR (95%CI)**	**p-Value**	**OR (95%CI)**	**p-Value**

Model 1	1.83 (1.20–2.79)	<0.001	1.10 (0.71–1.69)	0.123
Model 2	2.46 (1.50–4.03)	<0.001	0.68 (0.41–1.11)	0.123
Model 3	2.45 (1.46–4.11)	<0.001	0.57 (0.34–0.96)	0.033
Model 4	2.72 (1.56–4.76)	<0.001	0.63 (0.36–1.11)	0.111
Model 5	2.67 (1.53–4.68)	<0.001	0.66 (0.37–1.19)	0.167

Model 1 – HIV positive only or ART only as predictors. Model 2–HIV positive and ART as predictors. Model 3–HIV positive, ART, age and sex. Model 4–HIV positive, ART, age, sex, Blood pressure, Dyslipidemia, Diabetes Mellitus, Waist-Hip ratio, tobacco use, metabolic syndrome and Body Mass Index as predictors. Model 5–HIV positive, ART, age, sex, Blood pressure, Dyslipidemia, Diabetes Mellitus, Waist-Hip ratio, tobacco use, metabolic syndrome, Body Mass Index and either hs-CRP or Lp(a) as predictors.

## Discussion

### Summary of Results

We studied the distribution of Lp(a), hs-CRP and other traditional CVD risk factors among HIV positive (HIV+) patients on ART (HIV+ART+), HIV+ART– and HIV–ART– participants in the second largest teaching hospital in Ghana. We found that patients with HIV in Ghana had higher odds of having an elevated hs-CRP (>3mg/L) compared to healthy individuals without HIV. Similarly, compared with individuals who were HIV+ART–, we observed that individuals who were HIV+ART+ had a higher odds of elevated Lp(a). As expected, participants with diabetes mellitus, dyslipidemia, elevated waist-to-hip ratio, and metabolic syndrome were more likely to have higher hs-CRP levels, while participants with hypercholesterolemia and high LDL-C were more likely to have elevated Lp(a) levels.

### Comparison with Previous Research

Lipoprotein(a) and hs-CRP have been established as independent causal risk factors for atherosclerotic CVD in the general population [12] and are recognized in international cardiovascular clinical practice guidelines [20,21,22]. Findings from this study are consistent with data from other studies demonstrating high prevalence rates of inflammatory biomarkers such as CRP in PLWHA compared with controls [23]. PLWHA had significantly elevated hs-CRP and an elevated Lp(a) compared to controls, and this association remained after adjusting for age, sex, and other established CVD risk factors. Viral factors related to inflammation, other cytokines such as IL-6 [24] and differences in body composition [25] may partially explain this observation much the same way as potentially undiagnosed infections such as pulmonary tuberculosis and pneumonias may have also contributed to high hs-CRP levels in our study population [26]. This may have implications related to CVD events including myocardial infarction, heart failure, and peripheral artery disease because hs-CRP and Lp(a) are each associated with an increased risk of CVD independently of, but interacting with other CVD risk factors in both PLWHA [27] and the general population [28].

Among PLWHA, people taking ART had higher odds of elevated Lp(a). This finding corroborates findings from studies of PLWHA in Germany [29] and the United States [30]. In the US-based study, black PLWHA had significantly higher Lp(a) than white PLWHA, as did people with good HIV viral control and higher CD4 count. Enkhmaa et al. have posited that these findings may portend a modulation of Lp(a) risk properties (allele-specific apo[a] levels) by an improved HIV disease status that favours the production of smaller apo(a) size isoforms [30], resulting in elevated Lp(a) levels given its inverse relation to apo(a) size [31] This finding may have clinical implications, as studies have shown that prolonged usage of combination ART is associated with an increased risk of CVD [32] despite large HIV-related mortality benefits. The increased CVD risk may relate to some of these metabolic side effects that are mostly pronounced in patients treated with nucleoside/nucleotide reverse transcriptase inhibitors (NRTIs) or Protease inhibitors (PIs) [33]. With over 90% of HIV+ART+ patients on various combinations of NRTIs and PIs, deliberate efforts and interventions would have to be made to assess CVD risk factors routinely and to monitor patients to reduce the burden of CVDs and associated mortality if clinicians are to consolidate the gains made so far with ART. The net benefit of ART exceeds the risk of ART for PLWHA, both for HIV control and for reducing overall CVD risk [34]. Holistic CVD risk evaluation and monitoring with timeous interventions made can tilt this balance more favorably towards benefits.

### Strengths and Limitations

To the best of our knowledge this is one of the first studies to look at association between lipoprotein(a) and hs-CRP and how they relate to HIV, ART, and common CVD risk factors in west Africa. We investigated a relatively well-matched control population in terms of age and sex.

This study also has inherent limitations, including a non-representative sampling frame, its cross-sectional study design limiting causal inference, and its relatively small sample size.

## Conclusion

This study demonstrates that elevated levels of Lp(a) and hs-CRP are prevalent in PLWHA with differing associations with HIV and or ART use in Ghana. Potential clinical implications of the complex interactions of ART with CVD risk factors in this population warrant further study.

## Data Accessibility statement

Data can be accessed following a reasonable request to the SCRIPT study consortium.
